# Supplementary material for: Transcriptome-wide association study of attention deficit hyperactivity disorder identifies associated genes and phenotypes
Source: Nat Commun. 2019 Oct 1;10:4450. doi: 10.1038/s41467-019-12450-9 (PMC6773763; doi:10.1038/s41467-019-12450-9)
Supplement: Supplementary file 2 — Supplementary Information [file 41467_2019_12450_MOESM2_ESM.pdf]

Supplementary Figures/Tables

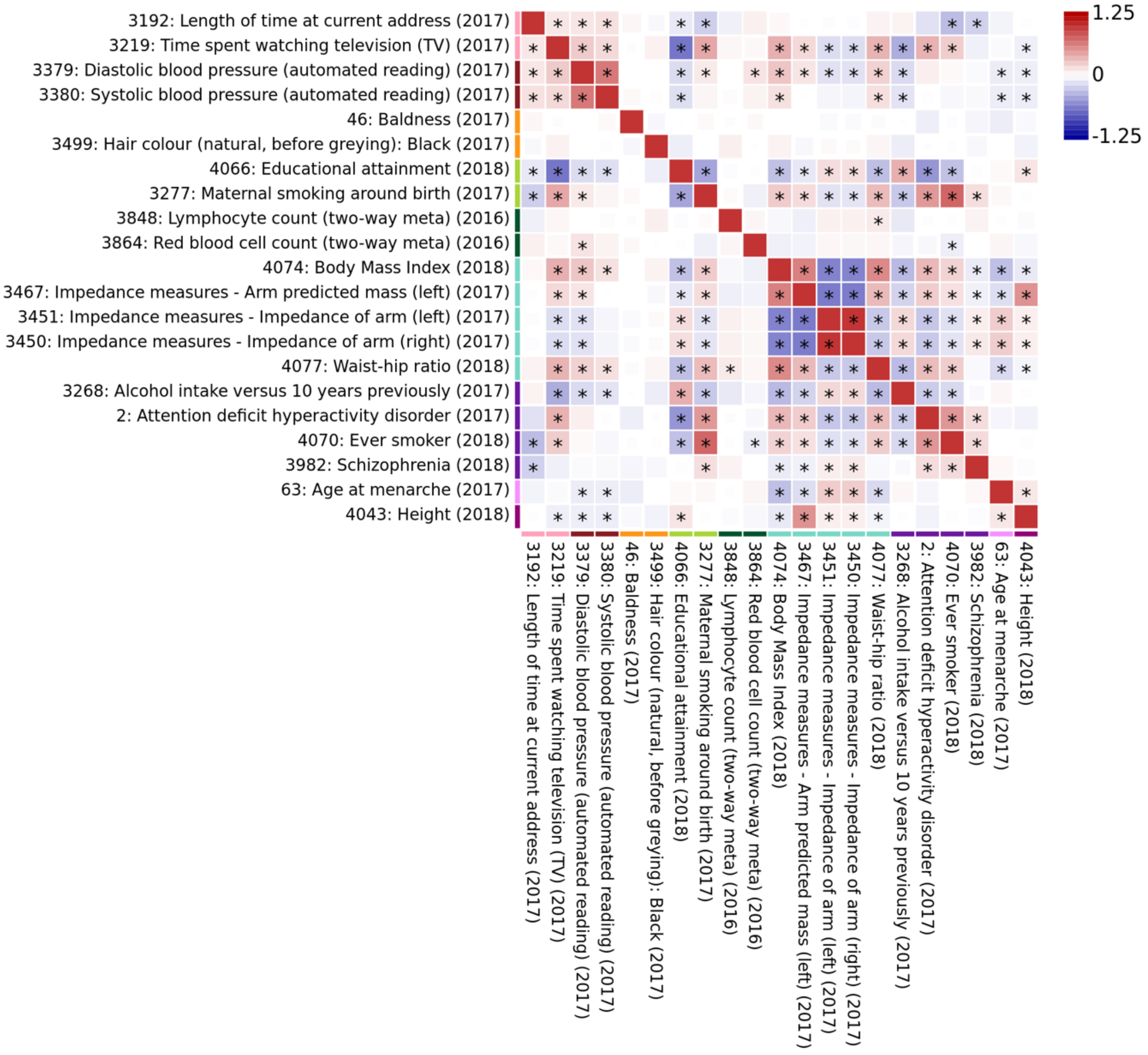

Supplementary Figure 1. Genetic correlation plot of phenotypes associated with top ADHD eQTLs. An asterisk in the box indicates the correlation passes Bonferroni significance threshold. Phenotypes are clustered by domain and derived from public genome-wide association study summary statistics.

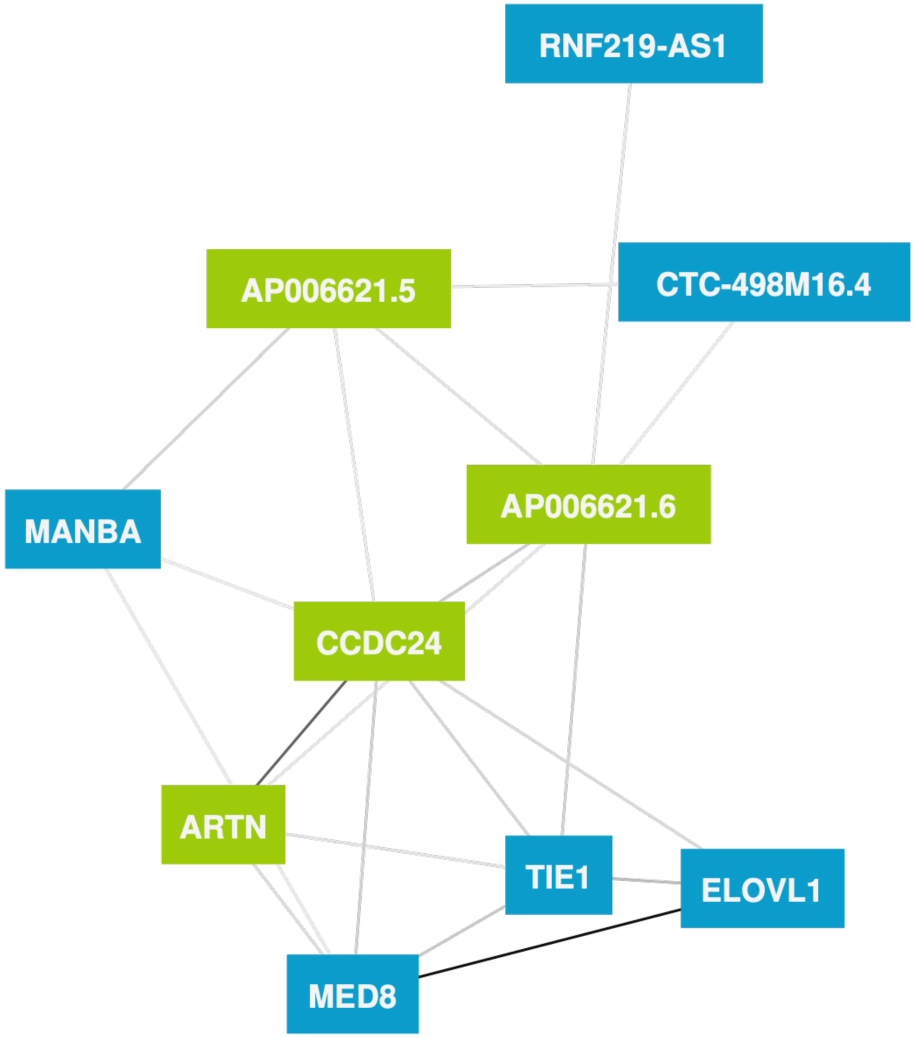

Supplementary Figure 2. Gene clustering of differentially expressed genes for the suggestive TWAS genes based on gene co-expression. (A) Public RNA sequencing data (N=31,499) was used to determine co-expression profiles. Gene cluster 1 identified in blue. Gene cluster 2 identified in green. Darker lines suggest a stronger co-expression.

Supplementary Table 1. Within-tissue panel significance thresholds

TI Panel	Number of genes	Within-tissue significance threshold
GTEx Brain Anterior Cingulate Cortex ba24	8731	5.73E-06
GTEx Caudate Basal Ganglia	9145	5.47E-06
GTEx Cerebellar Hemisphere	9451	5.29E-06
GTEx Cerebellum	10002	5.00E-06
GTEx Cortex	9162	5.46E-06
GTEx Frontal Cortex BA9	9031	5.54E-06
GTEx Hippocampus	8535	5.86E-06
GTEx Hypothalamus	8551	5.85E-06
GTEx Nucleus Accumbens Basal Ganglia	8913	5.61E-06
GTEx Brain Putamen Basal Ganglia	8759	5.71E-06
CMC DLPFC	10292	4.86E-06
Total	100572	4.9E-07

Supplementary Table 2. Significant pathways of TWAS genes identified through gene network analysis.

Pathway	Significance	Database
Dopaminergic neuron differentiation	2.0E-02	GO Processes
Protein serine/threonine phosphatase activity	4.3E-04	GO Function
Dendritic shaft	2.0E-03	GO Cellular

Neurotransmitter release cycle	3.6E-03	Reactome
Dopamine neurotransmitter release cycle	7.9E-03	Reactome
Highly calcium permeable postsynaptic nicotinic acetylcholine receptors	1.0E-3	Reactome
mRNA binding	4.1E-04	GO Function
Synapse assembly	5.6E-03	GO Processes

Supplementary Table 3. Phenotypes associated with top eQTLs derived from TWAS

**dbSNP or dbSNP ID**	**Phenotypes excluding ADHD (P-value)**
rs12741964	1. Red blood cell count (0.0001068091)<br>2. Educational attainment (0.000458)<br>3. Time spent watching TV (0.0003059)
rs2906457	1. Educational attainment (1.69E-10)<br>2. Alcohol intake versus 10 years previously (1.52E-8)<br>3. Cooked vegetable intake (8.41E-08)
rs223508	1. Lymphocyte count (2.21E-09)<br>2. Impedance of right arm (3.05E-09)<br>3. Impedance of left arm (5.89E-09)
rs1199039	1. Systolic blood pressure (9.24E-13)<br>2. Diastolic blood pressure (8.14E-12)<br>3. Height (3.6E-11)
rs10044618	1. Number of sexual partners (4.23E-12)<br>2. Educational attainment (6.08E-11)<br>3. Left arm predicted mass (1.2E-09)
rs3768046	1. Systolic blood pressure (7.61E-13)<br>2. Diastolic blood pressure (1.24E-11)<br>3. Height (9.1E-11)
rs223508	1. Lymphocyte count (2.21E-09)<br>2. Impedance of right arm (3.05E-09)<br>3. Impedance of left arm (5.89E-09)
rs11210892	1. Educational attainment (6.4E-19)<br>2. Age of menarche (5.47E-14)<br>3. Ever smoker (3.57E-13)
rs1410739	1. Hair colour (black) (0.00003779)<br>2. Baldness (0.000128406)<br>3. Hair colour (0.000128406)
rs12760274	1. Educational attainment (1.16E-09)<br>2. Maternal smoking around birth (4.37E-07)<br>3. Length of time at current address (0.000008061)

rs11246314	1. Waist-hip ratio (1.49E-13)<br>2. Impendance of right leg (2.35E-11)<br>3. Impedance of left leg (9.84E-11)
rs4963153	1. Waist-hip ratio (1.58E-13)<br>2. Body mass index (5.6E-09)<br>3. Impedance of right leg (6.64E-09)
rs6597981	1. Waist-hip ratio (8.69E-14)<br>2. Impedance of right leg (1.68E-10)<br>3. Waist-hip ratio (2.02E-10)
rs4963153	1. Waist-hip ratio (1.58E-13)<br>2. Body mass index (5.6E-09)<br>3. Impedance measures (6.64E-09)
rs12760274	1. Educational attainment (1.16E-09)<br>2. Maternal smoking around birth (4.37E-07)<br>3. Schizophrenia (0.00000905)
rs10902221	1. Waist-hip ratio (1.85E-13)<br>2. Impedance of right leg (3.22E-11)<br>3. Impedance of left leg (1.03E-10)
