# Supplementary material for: Transcriptome-wide association study of attention deficit hyperactivity disorder identifies associated genes and phenotypes
Source: Nat Commun. 2019 Oct 1;10:4450. doi: 10.1038/s41467-019-12450-9 (PMC6773763; doi:10.1038/s41467-019-12450-9)
Supplement: Supplementary file 4 — Description of Additional Supplementary Files [file 41467_2019_12450_MOESM4_ESM.pdf]

Title: Supplementary Data 1

Description: Contains the summary statistics for the transcriptome-wide association study
